# How Families Manage the Home Environment for Young People With Asthma and Allergic Sensitisation: A Qualitative Study

**DOI:** 10.1002/ppul.71013

**Published:** 2025-03-12

**Authors:** Grace Lewis, Linda Milnes, Jürgen Schwarze, Alexandra Adams, Alistair Duff

**Affiliations:** ^1^ School of Psychological Science University of Bristol Bristol UK; ^2^ Asthma UK Centre for Applied Research, USHER Institute University of Edinburgh Edinburgh UK; ^3^ School of Healthcare University of Leeds Leeds UK; ^4^ Child Life and Health, Centre for Inflammation Research University of Edinburgh Edinburgh UK; ^5^ Leeds Teaching Hospitals, NHS Trust Leeds UK

**Keywords:** children, grounded theory, house dust mite, indoor allergens, pets, qualitative

## Abstract

**Background and Aim:**

Children and young people (CYP) with severe, sub‐optimally controlled asthma and co‐existing allergic senitization to indoor aeroallergens, such as pet dander and house dust mite (HDM), would likely benefit from reduced allergen exposure. Multiple allergen remediation interventions exist and are often suggested to families in secondary care asthma clinics in the United Kingdom. Evidence suggests remediation uptake is low or partial but there is sparse evidence to explain why. This study aims to explain how families in this situation make decisions about home‐based allergen remediations.

**Methods:**

In‐depth qualitative interviews with CYP and mothers were analyzed, and a grounded theory approach was used to develop a theory to explain decision‐making processes and behaviors.

**Results:**

Ten CYP aged 11−15 years and 11 mothers were interviewed. The core finding was that families iteratively respond to changes in how certain they are in their asthma management decisions and actions. For allergen remediation uptake, this certainty varied depending on seeing an outcome‐exposure relationship, understanding asthma severity, variability, and asthma control at the time of remediation decision‐making. Understanding the mechanistic role of allergen exposures in asthma was challenging for families, and ongoing bi‐directional communication with clinicians was essential in supporting long‐term decision‐making.

**Conclusion:**

The theory explains the often elongated, reactive process of allergen remediation decision making and implementation. It also explains other elements of family management of asthma, and their interconnections. Families' iterative responsiveness suggests opportunities to intervene and promote earlier, preventative behavior change.

## 1 Background

Asthma affects over one million children and young people (CYP) in the United Kingdom (UK) [1], and severe asthma rates also exceed that of other European countries, particularly amongst 13–14‐year‐olds [2]. Prevalence of allergic senitization is rising, particularly in western Europe [3] with estimates of CYP with co‐existing asthma and allergic senitization ranging between 30% and 79% [4, 5, 6]. The mechanisms between indoor allergen exposure and adverse asthma outcomes are not fully understood [7] but are thought to involve type IVb (T2) immune responses [8]. While the extent of the contribution of allergic senitization and indoor allergen exposure to asthma attacks is uncertain, [9] home‐based house dust mite (HDM) levels correlate with children's asthma symptoms [10]. It is plausible that indoor allergen exposure and asthma symptoms and attacks are linked, [11, 12, 13] since exposure to allergens triggers bronchospasm and increases bronchial hyperreactivity in those sensitized; moreover cessation of such exposure can reduce asthma symptoms [14, 15, 16].

Self and family management of asthma is complex and often burdensome. Alongside medicating and monitoring asthma, [17] families of CYP with co‐existing allergic senitization can be advised to reduce allergen exposures in the home, and exposure to other potential triggers, such as environmental tobacco smoke (ETS) [18]. Evidence suggests that families rarely re‐home pets when advised [19, 20]. Several home‐based allergen reduction methods exist (such as air purification, HDM proof bedding, high efficiency particulate absorbing (HEPA) filtered vacuum cleaners and carpet removal), but evidence for these remains somewhat contentious due to limited evidence to support use, [21] and preference for meta‐analyzed systematic review data where trial heterogeneity limits this [22]. Yet, individualized practical allergen reduction advice for families is suggested, as evidence suggests overall effectiveness [23].

There is little evidence to suggest what influences whether advice is followed by families, and particularly parents' and CYP's perspectives on this. Often behavioral research focuses on irritant triggers, neglecting indoor allergens or CYP's allergic sensitivity status. Families living in areas of multiple deprivation remain under‐represented in this research area in the UK, [24] yet many of the interventions that may be suggested to families will incur out‐of‐pocket expenses. It is recommended that interventions promoting behavior change are evidence based, theory based and person‐centered [25, 26, 27, 28, 29] magnifying the importance of patient and family perspectives to inform interventions. The purpose of this study was to explain how families and CYP with severe and/or sub‐optimally controlled asthma and co‐existing allergic senitization to HDM and/or domestic pet dander, manage the indoor environment and what influences their avoidance uptake decisions and behaviors.

### 1.1 Study Design

A grounded theory approach was used to generate qualitative data through interviews, perform analyses, and develop an explanatory theory grounded in participants' accounts by discussing the meaning behind what was said [30]. Grounded theory is useful for theory development and areas scantly researched [31].

### 1.2 Recruitment and Sampling

Participants were recruited from a multi‐disciplinary hospital‐based severe and difficult asthma clinic in Yorkshire, UK. Clinicians (A.A. and A.D.) identified eligible patients for interview. Pre‐determined eligibility criteria informed by a review [24] directed initial purposive sampling (summarized in Table 1). Grounded theory includes theoretical sampling, whereby concurrent data generation and analyses inform subsequent sampling decisions. This included iterative alteration of interview questions (topic guide: supplementary file) [32, 33]. This process of sampling becoming increasingly purposive also involved considering adaption of inclusion criteria and seeks to maximize the potential transferability of the findings to wider groups with similar characteristics [30].

**Table 1:** inclusion criteria.

Inclusion	Exclusion
CYP aged 11–16 years	Other age groups: Over 17 s are likely to have begun transition to adult asthma care
Asthma severity: Asthma that is difficult to treat or sub‐optimally controlled asthma, defined by presence of one or more of the following‐ 1. 2 or more acute asthma attacks requiring medical attention within the last 12 months and/or 2. Regular asthma symptoms (e.g., cough, night‐symptoms, dyspnoea, wheeze) and/or 3. Over‐use of short‐acting beta‐agonist—(SABA‐a rescue/reliever inhaler), indicated by use of SABA to relieve symptoms more than twice per week [21]	Other co‐existing chronic respiratory conditions (e.g., Cystic Fibrosis, Bronchiectasis)
Co‐existing allergic senitization, defined by ≥3 mm (or greater) wheal on skin‐prick testing [21] for HDM and/or animal dander. (Other additional allergy or sensitivities will not lead to exclusions)	Those not meeting this criterion
Parent(s)/guardians/carers of CYP recruited (Carers will be classified as a main caregiver as identified by the parent/guardian)	Those not meeting this criterion
English language spoken	Those who feel unable to participate in interviews in English

### 1.3 Data Generation

Interviews were conducted online or by telephone. Participants selected the interview type (dyadic or individual) and one family member declining did not preclude participation of another.

### 1.4 Ethics

Adult and CYP patient and public involvement (PPI) meetings and consultation informed study planning, development of appropriate study information, consent and assent forms and lay summaries of findings. Informed consent was obtained for mothers, and CYP assent with parental consent was taken for those aged 15 years or younger. Safeguarding was planned in line with UK NHS guidance.

Participants were offered a gift voucher of nominal value to show appreciation for their time, whilst minimizing the risk of coercion [34]. As two female CYP participated, CYP are identified by age only and parents are identified by the age of their child (and a letter to show quotations are from a variety of participants) to further protect anonymity.

### 1.5 Data Analysis and Rigour

Analyses were initially inductive, beginning from the data, but later deductive and abductive. This allows back‐and‐forth analyses, each interview to inform the next, and re‐examination of former transcripts considering newly developing analytic findings [35, 36]. Although the analytic process is iterative and non‐linear this includes initial open, axial, selective coding, and development of a core category. Memo‐writing, field notes and diagraming are also revisited throughout to inform the analytic process [30, 35] alongside team discussions. Whilst rigour is often viewed as self‐contained within this methodology [30, 35, 37] attention to criteria for rigorous qualitative research was made and reflexivity supplemented analytic memoing to avoid undue influence [38] of the primary researcher (G.L.) and team, who came from a range of professional backgrounds.

## 2 Results

### 2.1 The Sample

Twenty‐one individuals participated, including 10 CYP and 11 mothers. Eight interviews with mother and child dyads, and five individual interviews were conducted between November 2021 and June 2022. Interviews lasted between 20 min (individual) and 1 h 7 min (dyadic) and were audio‐recorded (with permission) and transcribed.

A supplementary table provides participant demographic characteristics and indoor environmental management uptake and gives context for the qualitative findings.

### 2.2 The Grounded Theory and Qualitative Findings

**Figure 1:**
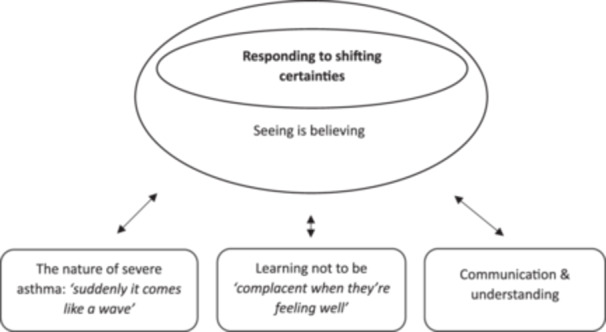
Responding to shifting certainties theory.

In depth qualitative analyses led to development of an explanatory theory, “responding to shifting certainties.” This was central in explaining beliefs and behaviors regarding indoor allergen and asthma trigger management. Moreover, it explains other beliefs and behaviors related to asthma self and family management via linked sub‐categories (Figure 1).

The theory explains how barriers and facilitators interconnect and may simplify or complicate decision‐making, depending on context, asthma symptoms and control at the time decisions are being made about indoor environmental remediation uptake.

Families experience tensions between the clarity and observability of trigger and allergen exposure‐outcome relationships, due to the variable nature of asthma and competing demands. These include managing family life, and managing asthma medication and monitoring, which often superseded consideration of triggers and allergens. However, families responded where levels of certainties shifted, for example, when medicinal treatments were adhered to and other explanations for reducing control or asthma attacks were absent, families then remediated allergens:

We got rid of all carpets, curtains, light shades, we wet dust, (he/she's) got anti‐allergy bedding… I got a de‐humidifier and a dust mite plug in. We did them (the remediations) all at once, this year when (CYP's name) were in hospital all that time…. and nothing were working and so that were the last resort. (Mother of 13(a)‐year‐old)

Responding to shifting certainties represents iterative, often reactive responses to changes in what is observed or felt and thus this core category is closely linked to the second category “seeing is believing.” Fully developed grounded theory explanations should be transferrable to explain a range of behaviors, [35] for example, early versus late allergen remediation adoption or non‐adoption, thus also accounting for cases which may differ to most in the sample, or “deviant,” negative cases [39, 40, 41]. Therefore, where a trigger or allergen cannot be seen or felt and linked to an adverse outcome, it is less likely families will believe they should respond with avoidance measures.

### 2.3 Seeing Is Believing

Families quickly identified the asthma triggers they were certain of, particularly exercise, ETS exposures, perfumes/sprays, and pollens. This certainty came from observing signs and feeling a difference in symptoms soon after exposure:

(after ETS exposure) a lot more coughing and a lot more phlegm. It's just bad for you, there are chemicals (13(b)‐year‐old)

One family noted:

We've got a wood‐burning stove, we use smokeless coal. I don't know if it's cos of the dry heat but if we have it on too much, (he/she) gets quite a quite chesty and we've stopped using it (Mum of 13(b)‐year‐old)

Linking exposure and symptoms led to avoidance, where possible. However, this connection and mechanistic understanding was not appreciated for indoor environmental allergens for CYP and most parents (all but one), which meant avoidance was delayed until there were no other observable explanations for periods of poorer control:

If we can't see it's (home pet exposure) making it worse. then I think we'd just carry on. (Mother of 13(d)‐year‐old).

Families were motivated by seeing the exposure‐outcome relationship and in‐turn, not seeing this presented a barrier to uptake of remediations. A process of eliminating other possible reasons for reduced control often left families viewing allergen remediation as a ‘*last resort*.’

(CYP's name) was just really poorly all the time, and not getting any better, so we just thought it's (HDM remediations) worth a try (Mother of 13(b)‐year‐old)

These barriers and motivators were inextricably linked to the following additional sub‐categories.

### 2.4 The Nature of Severe Asthma: “Suddenly It Comes Like a Wave”

The variability and episodic nature of asthma was linked by families to feeling partially resigned to unpredictability:

(his/her) asthma gets triggered even without those things. It can start anytime, anywhere (Mother of 15(d)‐ year‐old)

Conversely, those with co‐existing severe food allergies (requiring they carry an adrenaline auto‐injector) who are additionally under the care of an allergy clinic, reported feeling the need to control what they could to minimize observable risks and fear they associated with exposures:

I just don't know if it's (having a pet in home) going to make my asthma really bad… my asthma is bad enough already so I don't want anything else to make it worse (15(d)‐year‐old)

Amongst those with asthma and allergic senitization only, this episodic nature led some to question the impact of triggers and allergens, as even when remediated, unpredictability remained:

Sometimes I just get it randomly, like my asthma flares up randomly (15(a) years‐old)

it's a bit like a wave is (his/her) asthma, it'll be fine and then it'll change (Mother of 15(c)‐year‐old)

Participants frequently accepted and normalized ongoing baseline symptoms with peaks where asthma control deteriorated, and additional medications were required:

Yeah, sometimes I wake up with like coughing and sometimes it's hard to breathe, but if I take my inhalers, it settles down. (15(a)‐year‐old)

Similarly, allergic symptoms amongst those with un‐remediated homebased allergen‐exposures, were accepted and sat alongside apparent denial or under‐recognition that asthma control may also be affected by exposures such as pets CYP were sensitized and exposed to daily:

Mother of 13(d)‐year‐old: you sneeze a lot, don't you?

13(d)‐year‐old: yeah, and my eyes get swollen

Learning and understanding what a severe and/or uncontrolled asthma diagnosis meant occurred over long time periods. Some CYP were uncertain about their asthma severity, which surprised parents:

What do you mean you don't know? (Mother of 15(c)‐year‐old)

Severity was often confirmed by a severe attack requiring emergency medical care or explanations that highlighted CYP's vulnerability. Severity was seen as fluid, due to the relapsing‐remitting nature of symptoms:

I'd personally say it's moderate, it's not severe. We've only had 6 weeks where it's controlled but it makes a huge difference to my answer. Before they changed the inhaler (2 months ago) (he's/she's) had 6 lots of (oral) steroids within 5 months and was using the blue inhaler 6 or 7 times a day (Mother of 15(b)‐year‐old)

Understanding asthma severity and controllability with medication was learned over time and intertwined with decision‐making about allergen and trigger remediations.

### 2.5 Learning not to be “complacent When They're Feeling Well”: A Disparity Between Allergen Avoidance and Medication Adherence

Participants described patterns of learning the importance of medication adherence over time. Learning occurred through ongoing multi‐disciplinary team (MDT) advice, observations, and experiential feedback that once preventor inhaler use was consistently remembered, symptoms and control improved:

years ago, it used to be a big problem, like forgetting and stuff, but now that we're on top of it, all the time, it's gone really well (11‐year‐old)

Similarly, seeing a difference after taking medications was important in continuing adherence:

You do remember your montelukast every night, don't you? That's made a big difference (Mother of 13(c)‐year‐old)

These insights were closely connected to how families were influenced and reached decisions through their experiences, which provided feedback on how confident they were in following their management regimes:

probably one of the reasons (he's/she's) not taking it (cetirizine) is because subconsciously (he/she) doesn't think it does much…it's a battle I'm fighting, every time I ask it's “no” but it's because the asthma is controlled at the moment, so (he's/she's) likely to forget it (Mother of 15(c)‐ year‐old)

As learning the importance of medication adherence occurred over long periods, it was often prioritized over environmental management, albeit unintentionally.

### 2.6 Communication and Family Understanding: A Barrier and Facilitator

Families valued discussions with MDTs, particularly when decisions were shared and their opinions and experiences were valued, rather than feeling instructed by clinical advice:

You know, they didn't say we had to do this, but she said, it can be a lot better to have laminate, things like that, so yeah we did follow their advice (Mother of 13(a)‐year‐old)

Home visits by an asthma nurse to discuss asthma and managing the home environment were both welcomed and anxiety inducing for parents:

We had a home visit from somebody at Leeds… yeah, it was very nerve racking, it's like ‘ah no, what are they gonna find?' (Mother of 13(b)‐year‐old)

Those who described themselves as early adopters of indoor environmental remediations, would have preferred earlier referral for advice or primary care provision:

GPs could help a bit more in terms of finding out, you know send us to the hospital for proper check‐up, or give me a professional in asthma to check and do all sorts of investigations (Mother of 12(b)‐year‐ old)

For others, despite discussions with the MDT, families' own discussions and preferences would override clinical recommendations, where families could not clearly see an allergen exposure‐outcome relationship that took a similar pattern to viral or irritant triggers:

Mother (13 c): when we had allergy testing done, we'd only had our dog 6 months, then there's been no difference. We spent a lot of time talking about what we can do to help……we had a discussion obviously about the dog, about sleeping in bed with the dog (laughs) but I don't think that's ever going to change, is it? 13(c)‐year‐old: no, every day and I always will

Family understanding of how allergen exposures were potentially detrimental in those sensitized, was also limited and constrained by myths and misconceptions:

Int: Ok and do you think the dust is a problem or the dust mite allergy? Mother of 13b: I think dust definitely, when you're somewhere dusty it's worse, but I don't know…

Mother of 13(a): I didn't think that (he/she) would be allergic to dogs cos we'd had dogs all (his/her) life …. like I'm allergic to dogs but not my own… I think it means you get used to them

Family understanding of educational information and the challenges for them in relating this information to what they see and how CYP feel and interpret symptoms and control, often stalled remediation uptake.

## 3 Discussion

This grounded theory explains the iterative nature of developing sufficient certainty to influence remediation uptake. This occurs through repeated observations and by a process of eliminating factors apparently contributing to sub‐optimal asthma control and adverse outcomes. There is a gradual development of understanding asthma, its natural variability, individual severity, and the importance of adhering to medication, which feeds into decision‐making and prioritizing the timing of implementing components of self‐management. Grounding the theory in participants' accounts highlights that misconceptions and other experiences, such as home visits, also feed into decision‐making and may explain uptake variability.

These findings contribute to evidence gaps and explain low or partial uptake of allergen remediation advice in CYP with severe, sub‐optimally controlled asthma and senitization to HDM and pet dander [24]. Low or partial remediation uptake may be interpreted as low adherence to clinical advice. Theories and models explaining adherence in asthma self‐management often focus on medication adherence. This theory somewhat supports existing models explaining medication adherence with a necessity‐concerns approach [42]. Our participants also discussed balancing medication concerns with concerns about side effects and potential asthma outcome severity. This theory explained a similar pattern for allergy medication adherence and explains that multiple factors feed into allergen remediation decisions and behaviors in CYP and family management.

Iteratively developing certainty may conversely suggest periods of uncertainty. Uncertainty in living with and managing chronic conditions is well documented. Uncertainty in illness theory explains how those with chronic conditions and their caregivers understand the uncertain course their condition may take [43, 44]. In an extension of earlier work on uncertainty in illness theory, a qualitative study with families of children experiencing cystic fibrosis diagnostic investigations, suggested uncertainty led to precaution adoption [45]. This was somewhat true in our theory for those with co‐existing food allergies, for whom it is proposed the accompanying fear and anxiety described and perhaps access to allergists explained these differences. However, for most families in our study, a reactive approach was taken when other possible causes of deteriorating asthma were (self) eliminated. Our study goes some way to address the absence of qualitative research directly including CYP with asthma and allergic senitization in studies about illness uncertainty. In studies of families with a child with asthma, uncertainty is known to increase when asthma is exacerbated, [46] and familial uncertainty surrounds asthma etiology, symptoms, and consequences, particularly for parents [47]. However, our theory emphasizes that certainty is fluid and shifts with context, thus observations can lead to reactive self‐management behavior change. This theory may therefore be used as a precursor for development or updates of current educational or behavior change interventions promoting allergen remediation. First, these findings show limited family understanding of the mechanisms by which allergen exposures may reduce asthma control and that this may not translate into the same observational outcomes as viral or irritant triggers for CYP with asthma. Our participants also did not recognize that triggers and allergens may compound, particularly in tandem with viral infections [12]. Second, participants did not understand the concept of the unified airway [48]. Third, in an extension of this work, the findings outlined in this paper have been mapped to behavior theory [49]. Current behavior in this sample mapped closest to deconditioning, or rather, “Letting people experience a lack of reinforcement or even negative outcomes of the undesired behavior” [28, pp. 9], albeit an unplanned experience in participants, who had received allergen remediation advice. To move toward preventative remediation implementation in those who stand to benefit, our findings suggest the need to address misconceptions and alter beliefs hindering uptake. Relevant behavior change theories include precaution adoption approaches, self‐monitoring and self‐regulation. Self‐regulation has been noted for use in promoting asthma self‐management, [42, 50] but without specific focus on indoor allergen remediation. Finally, the frequent overestimation of asthma control and under‐recognition of severity has been reported for parents [51]. Our study supports that there is a notable disparity in comparing CYP's and parents' control and severity descriptions to accounts of signs and symptoms, and this was often a factor contributing to delayed remediation uptake.

There are strengths and limitations to this study. Adhering closely to the selected methodology allowed us to strive for theoretical saturation [30]. However, this concept is open to criticism since it is challenging to define or demonstrate [52]. Once saturation was suspected, a further three interviews were conducted allowing greater data interrogation and theory refinement. The constant comparative method and theoretical sampling [30] alongside team discussions, were vital in maximizing saturation likelihood, or rather achieving theoretical sufficiency [53] to allow theory development.

Our sample is dominated by mothers and male CYP. As mothers were primary caregivers in these families, this is not unexpected. The preponderance of male CYP may reflect that wheeze and asthma are more prevalent in boys, at least until adolescence [54] and males have significantly higher rates of allergic senitization to indoor aeroallergens into early adulthood [55]. Our sample goes some way towards addressing a gap in knowledge from families living predominantly in areas of multiple deprivation, since many (58%) lived in areas designated most deprived [56]. The decision to conduct interviews in English only was taken due to both practicalities, such as preparing study information in a variety of languages, and complexities of conducting interviews with interpreter; whereby the meaning of interview questions and answers can be altered through language interpretation, which may in‐turn affect credibility of qualitative analysis [57]. Exclusion of those unable to participate due to languages spoken may neglect potentially different perspectives and care needs and may limit transferability of our findings.

Future work could focus on intervention development to address the issues described. This could be enhanced by using the behavior change theory outlined. Participatory approaches to ensure interventions are suited to families who would stand to benefit from implementing them could be considered.

## 4 Conclusions

To our knowledge, this study is the first to explain beliefs and behaviors relating to indoor allergens in sensitized CYP with severe and sub‐optimally controlled asthma and their parents. It has afforded a theory of *responding to shifting certainties*, grounded in participants' accounts. It suggests families are likely to benefit from further interventions to address myths and misconceptions contributing to limited understanding of the pathophysiological role of continual home‐based allergen exposure in cases of sub‐optimally controlled asthma with allergic senitization. The theory explains decision making and uptake of interventions by encompassing multiple related factors. Therefore, these findings may be transferrable to other age groups, asthma severities and potentially other chronic conditions requiring multi‐faceted self and family management.

## Author Contributions

**Grace Lewis:** conceptualization, investigation, writing – original draft, methodology, writing – review and editing, formal analysis, project administration, data curation. **Linda Milnes:** conceptualization, funding acquisition, methodology, writing – review and editing, supervision, formal analysis. **Jürgen Schwarze:** conceptualization, funding acquisition, writing – review and editing, supervision. **Alexandra Adams:** funding acquisition, project administration, supervision, conceptualization, writing – review and editing. **Alistair Duff:** conceptualization, funding acquisition, methodology, writing – review and editing, formal analysis, supervision, project administration.

## Ethics Statement

The study was approved by a National Health Service (NHS) Research Ethics Committee (REC) and the Health Research Authority (REC:21/SW/0034) in the United Kingdom. Informed consent was obtained from participating adults. Participants under the age of 16 years gave informed assent, and a parent provided informed consent on their behalf.

## Conflicts of Interest

The authors declare no conflicts of interest.

## Supporting information

Supporting information.

Supporting information.

## Data Availability

The data that support the findings of this study can be made available upon reasonable request to the corresponding author.
