# Supplementary material for: How Families Manage the Home Environment for Young People With Asthma and Allergic Sensitisation: A Qualitative Study
Source: Pediatr Pulmonol. 2025 Mar 12;60(3):e71013. doi: 10.1002/ppul.71013 (PMC11898542; doi:10.1002/ppul.71013)
Supplement: Supplementary file 1 — Supporting information. [file PPUL-60-0-s002.docx]

**Interview topic guide**

**Background**

- Household composition/living situation. Location (rural/urban?)
- Time spent in the home and day-today activities

**Asthma**

- Can you tell me about having asthma / your child having asthma?

[Probes if needed: severity? Frequency of attack/last/most recent attack? Reliever inhaler use frequency? Regularity of emergency care visits and hospital or primary care attendances? Who takes the lead in the family for managing your/your child’s asthma? Do you wake up in the night because of your asthma?/ do you cough in the night or wake up with a tight chest or find it hard to breathe? Are you/is your child able to do the things you would like to do without asthma stopping you? How do you/ does your child find tasks like walking upstairs or taking part in sports at school? How do you find remembering your/your child’s medications? Can you tell me more about when/why it can be difficult to take asthma medications?]

**Asthma triggers**

- Can you tell me about anything you notice that makes your/your child’s asthma worse, or sets it off?

[Probes: It can help to think about things in the air, sometimes things you can see or things you can smell. Others might be things you have been advised to avoid by doctors or nurses (if participants have taken photos/drawn a picture this can be discussed here). Why do you think these might be triggers for you? -Do you have an asthma action plan? If so, do you have triggers written there? Do you talk about triggers at home? ]

- Can you tell me *how* or *why* you think those things trigger or set off your/your child’s asthma? It is ok if you do not know.

**Allergic sensitisation**

- Can you remember whether you/your child have had a skin prick test at a clinic/hospital? What did you think about the results/can you remember your results? [Probes: What did you think about the results when you were told? (if needs further probe- was it what you expected/were you surprised? Why is that?). Did doctors/nurses talk to you about it? Did the results make you think any differently about asthma triggers and allergens or did you stay the same? (can you tell me why?) How about others at home- were they surprised / did they change anything after the results?]
- [later addition after initial interviews] Do you think the things shown on your skin prick test / the positive results trigger your/your child’s asthma in the same way as other things you mentioned (for example, do you think house dust mite might affect your asthma in the same way as cigarette smoke or having a cold does? It is ok if you haven’t ever thought about this before) [Further Probes: do you have any ideas why/why not/ why it is the same/similar or different?]
- Do you/your child have any other allergies, such as food allergies? Can you tell me about how you manage those? [Probes: is there anything that makes it easy / difficult to manage these allergies?]

**Behaviour change/future intervention needs**

- Do you think it helps to avoid triggers and/or allergens or use extra care [probes: measures such as things they may have mentioned earlier, parent/family cleaning, not letting pets in certain rooms]?
- Do you think more of this would help control your asthma better? If not, can they explain why they think that?
- What kinds of things might help you to avoid triggers and/or allergens? Or help you to recognise them if there is difficulty with that?
- What might encourage you to avoid triggers and allergens (for example pets if pets are kept)?
- Do you feel in control of triggers and allergens or what support might help you feel you have better control?
- Is there anything else that affects whether you avoid triggers and allergens or not, that we have not covered yet?
- If we were to look at ways to help families reduce triggers and allergens at home, what do you think would be helpful? [Probes- If there is an information need, what formats would be most useful, delivered by whom? If you were to advise someone in your position or another parent of a child/young person with asthma, about avoiding triggers and allergens, what advice would you give?]
