# Supplementary material for: How Families Manage the Home Environment for Young People With Asthma and Allergic Sensitisation: A Qualitative Study
Source: Pediatr Pulmonol. 2025 Mar 12;60(3):e71013. doi: 10.1002/ppul.71013 (PMC11898542; doi:10.1002/ppul.71013)
Supplement: Supplementary file 2 — Supporting information. [file PPUL-60-0-s001.docx]

Supplementary table showing CYP participant age, residential multiple deprivation index (MDI: Ministry of Housing, Communities & Local Government, 2019^56^) decile, remediations undertaken (X), and ongoing exposures (shown as blank) (-- = removed: ++ = frequently): table from *Lewis, (2022)^49^*

| CYP age years (& ID) | MDI decile | Allergen sensitisations | Allergy | Continued exposures at home (pets/ETS) | Air purifier | Avoids Foods | --Soft Furnishing removal | Carpet removal | HDM plug in | De-humidifier | Allergy bedding | Stopped ETS | Extra cleaning | Hot wash (bedding) | Vacuums ++ | Freezes soft toys | Handwash | Avoid pets | Treat mould | Temp control | Tumble dries | Ventilates | --Soft toys | 100% cotton bedding | No bunkbed | Pet removed |
| --- | --- | --- | --- | --- | --- | --- | --- | --- | --- | --- | --- | --- | --- | --- | --- | --- | --- | --- | --- | --- | --- | --- | --- | --- | --- | --- |
| 15a | 1 | HDM, cats, dogs, pollen | Yes (foods) | No | X | X |  |  |  |  |  |  |  |  |  |  |  |  |  |  |  |  |  |  |  |  |
| 13a | 1 | HDM, cats, dogs, pollen | No | Dogs |  |  | X | X | X | X | X | X | X |  | X |  |  |  |  |  |  |  |  |  |  |  |
| 11 | 8 | HDM, cats, dogs, pollen | No | Dogs (father’s home) |  |  |  |  |  |  | X |  | X | X | X |  |  |  |  |  |  |  |  |  |  |  |
| 13b | 5 | HDM, cats, pollen | No | No |  |  | X | X |  |  |  |  | X |  | X |  |  |  |  |  |  |  | X |  |  |  |
| 13c | 1 | HDM, cats, dogs, pollen | No | ETS unclear; co-sleeps with dog |  |  |  |  |  |  |  |  | X | X | X | X |  |  |  |  |  |  | X |  |  |  |
| 12a | 1 | HDM, cats, dogs, pollen | Yes (foods) | No |  | X |  |  |  |  |  |  | X | X |  |  | X |  |  |  |  |  |  |  |  |  |
| 15b | 1 | HDM, cats, dogs, pollen | No | No |  | X |  | X |  |  | X |  | X |  |  |  | X |  |  |  |  |  |  |  |  |  |
| 13d | 2 | HDM, cats, dogs, pollen | No | Cats, dogs |  |  |  |  |  |  |  |  |  | X |  |  | X |  |  |  |  |  |  |  |  |  |
| 12b | 1 | HDM, cats, dogs, pollen | Yes (foods) | No |  | X |  | X partial |  | X |  |  |  |  | X |  |  | X | X | X | X | X | X | X |  |  |
| 13e | 6 | HDM, cats, pollen | No | No |  |  |  |  |  |  |  |  | X | X | X |  |  |  |  |  |  |  |  |  | X | X |
| 15c | 3 | HDM, cats, pollen | No | No |  |  |  | X |  |  | X | X |  |  |  |  |  |  |  |  |  |  | X |  |  | X |
| 15d | 1 | HDM, cats, dogs, pollen | No | No |  |  |  |  |  |  | X |  | X |  |  |  |  |  |  |  |  |  |  |  |  |  |
